# Non-Intrusive Fish Weight Estimation in Turbid Water Using Deep Learning and Regression Models

**DOI:** 10.3390/s22145161

**Published:** 2022-07-10

**Authors:** Naruephorn Tengtrairat, Wai Lok Woo, Phetcharat Parathai, Damrongsak Rinchumphu, Chatchawan Chaichana

**Affiliations:** 1 School of Software Engineering, Payap University, Chiang Mai 50000, Thailand; naruephorn_t@payap.ac.th (N.T.); phetcharat@payap.ac.th (P.P.); 2 Department of Computer and Information Sciences, Northumbria University, Newcastle Upon Tyne NE1 8ST, UK; 3 Department of Civil Engineering, Faculty of Engineering, Chiang Mai University, Chiang Mai 50200, Thailand; damrongsak.r@cmu.ac.th; 4 Department of Mechanical Engineering, Faculty of Engineering, Chiang Mai University, Chiang Mai 50200, Thailand; chatchawan.c@cmu.ac.th

**Keywords:** non-intrusive methods, aquaculture, weight estimation, deep learning, machine vision

## Abstract

Underwater fish monitoring is the one of the most challenging problems for efficiently feeding and harvesting fish, while still being environmentally friendly. The proposed 2D computer vision method is aimed at non-intrusively estimating the weight of Tilapia fish in turbid water environments. Additionally, the proposed method avoids the issue of using high-cost stereo cameras and instead uses only a low-cost video camera to observe the underwater life through a single channel recording. An in-house curated Tilapia-image dataset and Tilapia-file dataset with various ages of Tilapia are used. The proposed method consists of a Tilapia detection step and Tilapia weight-estimation step. A Mask Recurrent-Convolutional Neural Network model is first trained for detecting and extracting the image dimensions (i.e., in terms of image pixels) of the fish. Secondly, is the Tilapia weight-estimation step, wherein the proposed method estimates the depth of the fish in the tanks and then converts the Tilapia’s extracted image dimensions from pixels to centimeters. Subsequently, the Tilapia’s weight is estimated by a trained model based on regression learning. Linear regression, random forest regression, and support vector regression have been developed to determine the best models for weight estimation. The achieved experimental results have demonstrated that the proposed method yields a Mean Absolute Error of 42.54 g, R2 of 0.70, and an average weight error of 30.30 (±23.09) grams in a turbid water environment, respectively, which show the practicality of the proposed framework.

## 1. Introduction

Thailand’s economy has been generally based on agriculture production, with the sector employing around one-third of the country’s labour force. Aquaculture production in Thailand has continuously increased since 1995 [1]. Fish are a healthy food that are an excellent source of protein, minerals, and essential nutrients. This leads to enormous demand that exceeds the production capacity. Therefore, the development of fish farming with modern technology will improve fish monitoring operations to efficiently feed and harvest fish, while also being environmentally friendly. In addition, non-contact measurements of fish body size and weight will reduce stress and injury to the fish. This research is the first step of modern fish farming in Thailand to measure fish weight by non-intrusive methods. Modern aquaculture has rapidly developed in recent years. Extensive expansion of traditional aquaculture has resulted in it being transformed into modern 5G aquaculture by automatic and precise task-based machines. The machines perform classification, prediction, and estimation, and have many benefits, including reducing operational time.

Fish weight estimation is one of the most challenging problems in aquacultural applications. Recent methods of fish weight estimation have been proposed, as in Ref. [2], which is comprised of three components: the simplified VGG module, the multi-dilated convolution module, and the squeeze-excitation module (SE). In Ref. [3], the fish biomass estimation has employed an Arduino board to measure live-fish weights for in-land facilities or offshore cages. Fish size is a crucially essential parameter for estimating fish weight through different growth stages. Machine vision provides an automatic and effective approach for measuring size where special cameras are required for capturing free-swimming fishes. For example, stereovision system [4] stereo cameras have been used for distance measurements and the capture of fish in a tank, which the CNNs method use for fish detection and a regression method predicts the fish’s weight. Stereo cameras were set for capturing images of fish. The Nile Tilapia weight prediction method performs fish detection via image processing techniques and depth calculation, which is given by stereo frames through disparity values. The length estimation was estimated from the contour of the fish, then it is converted into pixel length in metric units by using disparity information. Polynomial regression was used for computing the weight of fish given by the estimated length of the fish. The strength of the regression principle is the simplicity of development and low computational complexity. Six cameras were set at a fixed distance—with three being near-infrared cameras and three being general cameras [5]—where a deep convolutional neural network (DCNN) estimated fish weight from the length, weight, and girth of the fish. The residual neural network (ResNet) and LinkNet for segmenting fish images then estimate the weight from the area of the detected fish [6]. Machine learning approaches for predicting animal weight can be categorized into two groups, which are regression learning and deep learning. The regression approach has been broadly used to develop models for the prediction of body weight. Regression learning for weight prediction requires animal features that are significantly related to weight to be used for learning. Thus, animal feature identification is essential to a model for learning and accurately predicting the animal’s weight. For example, thirty attributes of sheep are used from images, i.e., shape, size, and angles with k-curvature, in Ref. [7]. Eight regression models were used and extracted for the regression models. These are linear regression (LR), support vector regression (SVR), K-neighbors regression (KNR), multi-layer perceptron regression (MLPR), light Gradient boosting machine (GBM), extreme gradient boost regression (XGBR), Gradient boosting regression (GBR), and random forest regression (RFR). The research found that RFR yields the best result with an *R*^2^ at 0.687. In Ref. [8], the weight-prediction-method-based classification and regression tree for goats was proposed and given by seven morphometric traits, i.e., body length, heart grith, rump height, rump width, ear length, cannon circumference, and head width—and including age and sex. The results indicated that sex, heart girth, and age are highly correlated with variations in the body weight of goats. In Ref. [9], the state-of-the-art regression models from SciKit-Learn were employed to predict the body weight of Hereford cows and were given by 12 body size measurements (withers height, hip height, chest dept, chest width, width in maclocks, sciatic hill width, oblique length of the body, oblique rear length, chest girth, metacarpus girth, and backside half-girth) and age (full years). The paper found that RFR yields the best weight prediction of Hereford cows at a 0.644 regression score (*R*^2^). In Refs. [10,11], only three attributes of sheep—body length, body height, and chest girth—were provided for predicting sheep. The weight-prediction methods were computed by a multiple linear regression analysis and generalized linear model. The accuracy performance of the model has an *R*^2^ score of 0.62. 

A deep learning approach is currently the favored method for handling complex data, such as that in an underwater environment. Deep learning is a non-linear approach for unsupervised or supervised learning. A deep learning framework [12] is composed of two sections where the first section is convolutional neural networks (CNNs) and the second section is a full-connected multi-layer perceptron (MLP). The CNNs transform input data into multiple levels of representation to extract significant spatial information from the input data by performing convolution functions, pooling functions, and activation functions, respectively. The significant features of input data will automatically be discovered by the CNNs section. A pooling function is used to reduce the number of parameters by using masking and mathematical operations, i.e., the maximum, the average, the weighted average, and the *L*_2_ norm, which is used to select the represented parameter from the masking. A sparsity vector will be obtained as the results of the CNNs then pass through the fully-connected MLP. The MLP process consists of two dense layers that estimate event activity probabilities for each frame. A softmax function is last and is used as an activation function to classify the sound into its corresponding class. The softmax function is considered the generalization of the logistic function, which aims to avoid overfitting. An advantage of deep learning methods is that they do not require feature extraction for an input sound. Deep learning has been extensively employed in aquaculture for example detection, classification, counting, monitoring behavior, or defect detection. Real-time object detection methods such as YOLO (You Only Look Once) [13,14] and COCO (Common Objects in COntext) [15] were introduced to detect aquatics, for example, the DeepFish method in Ref. [16], which analyzed remote underwater fish habitats. The YOLO algorithm is formulated as a regression problem and provides the class probabilities for image detection. The YOLO framework is based on convolutional neural networks (CNN), which requires only a single forward propagation through a neural network to detect objects. The YOLO algorithm works using the following three techniques: Residual blocks, Bounding box regression, and Intersection Over Union (IOU). YOLO yields superior performance over the other object-detection techniques. Deep learning has also been integrated with traditional methods into a myriad of applications that can be used for a variety of purposes. For example, DeepFish with a support vector machine (SVM) method in Ref. [17] is used for the recognition of fish from 23 fish species from a video captured by underwater cameras in the open sea. The deep learning architectures of DeepFish-SVM are constructed by two convolution layers, a non-linear layer, a feature pooling layer, a spatial pyramid pooling, and an SVM classifier. Image augmentation was used to enlarge the training set for the species whose image number is less than 300. The accuracy results of DeepFish-SVM are compared to DeepFish-Softmax and Deep-CNN. DeepFish-SVM yields slightly better results than the rest. In Ref. [18], the proposed method based on SVM and CNNs is applied for classifying regional areas of four crops (paddy rice, potatoes, cabbages, and peanuts), roads, and buildings from remote-sensing images. The SVM process handles pixel-based classification while the CNN process performs block segmentation to enhance the classification results. The proposed method yields the high accuracy performance of regional area classification. In Ref. [19], the weighing of heifers is introduced by using the Mask-RCNN segmentation algorithm with a proposed CNN-based mass prediction model. In addition, a pig contactless weight system is presented in Ref. [20] which uses the pig-detection-based CNNs and the weight regression model. Three-dimensional (3D) cameras were used for capturing posture images. The weight of pigs is estimated from the back of pigs in top-view depth images. Other methods of weight-estimation-based 2D and 3D reconstruction have recently been proposed. Furthermore, deep learning was used for predicting the weight of cattle in Ref. [21]. Deep learning extends to perform the regression task with automatic feature extraction given by 2-dimensional images. Individual cattle were captured through the water trough platform that provides a cattle’s ID, images, time, and weight. Three types of convolutional neural networks (CNN) with various regularization functions were established to determine the best methods, which are combination recurrent neural networks (RNN)/CNN with and without attention, recurrent attention model without CNN, RestNet 8, and EfficientNetB1. The Adam optimizer with a learning rate of 0.005 was set for training the models for 10 epochs and at a batch size of 32 to 256. The experimental results showed that the RNN/CNN model achieved the highest performance among the rest models at a Mean Absolute Error (MAE) of 23.19 kg. The volume and weight estimation of an apple was proposed in Refs. [22,23] by simulating 3D images using a single multispectral camera and near-infrared linear-array structured light. Height features were mapped via 2D and 3D reconstruction images. The PLS and LS-SVM were employed to estimate the volume and weight of apples. A 3D image can be directly obtained via special cameras, for example, binocular stereo cameras, a laser-based camera, or an RGB-depth camera that generates the spatial information of the X-Y dimension with the height information of Z represented.

Machine learning approaches for weight prediction can be categorized into two groups, where the first group is based on the regression approach and the second one relies on a deep learning approach. The regression approach takes advantage of the simplicity and fast performance but requires the feature extraction process. The selected features are vital and significantly affect the prediction performance. Generally, the regression approach needs more than five features. Therefore, feature acquisition is still a challenging issue, and is time- and cost-consuming. On the other hand, deep learning approaches deliver a compact algorithm given by input images and then return a result. However, deep learning approaches usually require special cameras with high computational complexity for weight estimation. A captured image by an underwater camera is influenced by complex non-linear factors due to luminosity change, turbidity, various backgrounds, and moving aquatic animals. Underwater monitoring is one of the most challenging problems due to uncertain environments caused by changes in illumination and shadow, turbidity, underwater–aquatic confusion, camera limitations, and moving aquatics. These result in the low quality of image capture. Therefore, the practicality of a fish weight-prediction method for turbid water that can be used in real fish-farming applications is still an open problem.

The present paper proposes a novel low-cost practical single sensor imaging system with deep and regression learning algorithms for the non-intrusive estimation of Tilapia weight in turbid water environments. The proposed method brings new contributions. Firstly, only a low-cost single camera is required for observing the underwater fish (no other special equipment or sensor is used for monitoring fish). Thus, the fish are not injured during the weighing process, which is beneficial to the health of the fish. Secondly, the proposed method can determine the fish’s weight in the turbid underwater environment. For turbid water, the proposed method can process the video frames with or without an image-enhancement process. This flexibility favors practicality in real fish-farming applications. Only as little as three attributes are required for predicting the fish’s weight: (i) fish’s age, (ii) the length and width of the fish, and (iii) the depth between the fish and the camera. These attributes are automatically computed by the proposed algorithm in one-go. Finally, the proposed method is computationally simple and comprises two major steps, i.e., Tilapia detection-based deep transfer learning and Tilapia weight estimation-based regression learning. This augments the proposed method with low computational time and thus results in faster execution. The proposed machine learning models are amenable to interpretability by the users. For example, once the fish is detected, the estimated length and height of the fish, as well as the depth information from the camera, are made known to the user. By manually inputting the age of the fish by the user, the user will be able to determine the weight of the intended fish.

This paper is organized as follows: Section 2 presents the machine vision algorithm to estimate the weight of Tilapia in an underwater environment. Next, Section 3 evaluates and elucidates the performance of the proposed Tilapia weight estimating algorithm. Finally, Section 4 summarizes the proposed estimation method and future research prospects.

## 2. Methodology

**Figure 1:**
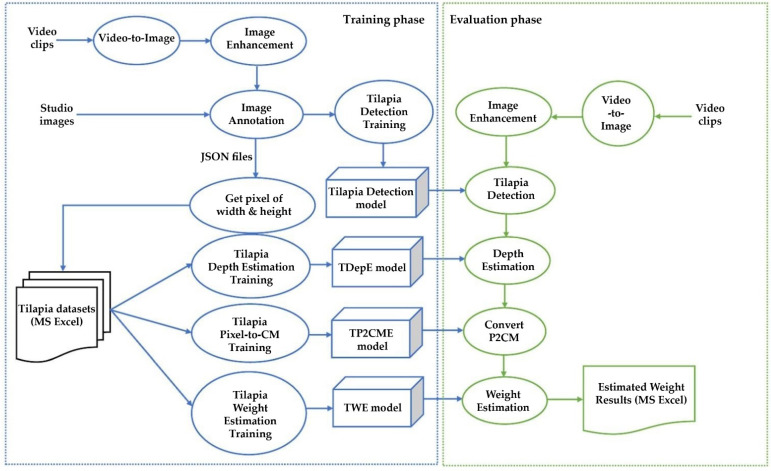
Proposed TWE-DRL algorithm.

The proposed method combines two steps: a Tilapia detection step and Tilapia weight-estimation step. The proposed method was started by training the models and then using the trained models in an evaluation phase. The training phase performs data preparation for Tilapia Detection Training and then generates a Tilapia detection (TDet) model that is based on deep transfer learning. In the Tilapia weight-estimation step, three models are trained by using regression learning. These models are are Tilapia depth estimation (TDepE), Tilapia pixel-to-centimeter estimation (TP2CME), and tilapia weight estimation (TWE). Therefore, the proposed algorithm is named Tilapia weight estimation—i.e., the deep regression learning “TWE-DRL” algorithm. The algorithm of the proposed method is illustrated in Figure 1. 

The input parameters of the TDepE model consist of the age of the fish and the fish’s length and width in pixel units. In the process of data acquisition, the ages of the fish were recorded along with the fish-image capture every two weeks during the feeding process. The actual length and width of the fish were obtained by manually extracting this information from the image-annotated labels of the fish. Therefore, the training dataset of the TDepE model contains the actual values of the fish’s age, length, and width. In practice, the age of the fish will be obtained from a fish farmer with prior knowledge. The input parameters of the TP2CME model use the same parameter set as TDepE and add the distance between the fish and the camera with regards for the depth parameter. The depth dataset contains three independent attributes, which are the age, the fish’s length and width in pixel units, and the depth. Firstly, depth information acquisition was manually determined by humans. There are stripes on the ground and indicated sides from the front of the camera to the end of the tank. Each strip is 10 cm apart from one another. Strips are used as a reference distance from the camera. Hence, the fish’s distances were estimated in response to the nearest band where the fish was located. The depth of the fish affects the size of the fish, i.e., when a fish is close to a camera then the depth is small, and the length and the width of the fish are larger when it is further away. The input parameters of the TWE model follow the same steps as the TP2CME model where the output of the TP2CME model is an independent parameter of the TWE model plus all of the independent parameters from the TP2CME dataset. For the TWE training dataset, the actual length and width of fish was provided from Studio photography. The details of each individual step are elucidated in the following sections.

The proposed TWE-DRL algorithm has two major processes, which are to detect and extract the size of an individual Tilapia in an image and to estimate the depth of the fish from the camcorder, then convert the size of the Tilapia from pixels to centimeters given the estimated depth. Finally, the weight of the Tilapia is predicted from the fish’s size with the inclusion of the fish’s age in weeks. In order to achieve these goals, four-training models are required and named TDet, TDepE, TP2CME, and TWE. The details of each individual step are elucidated in the following sections.

### 2.1. Tilapia Detection

Tilapia-detection-based deep transfer learning is used to create a model for detecting Tilapia in digital images. Tilapia detection is established through deep learning networks as their backbone and the detection network is used to extract features from the input images and localization, respectively. An object detection approach can be categorized into two types, i.e., one-stage detectors and two-state detectors. One-stage detectors use a single network to predict object bounding boxes from images directly then classify the probability scores from the images—for example, YOLO, SSD, and RetinaNet.

Two-stage detectors mark regions of the target instead of learning from the whole image. Next, the proposal regions will be passed into a classifier and regressor, respectively. Region Proposal Networks (RPNs) are used for searching possible target regions as the first stage. The second stage extracts significant features by using a region-of-interest (RoI) pooling operation from individual candidate regions for the following classification and bounding-box regression. Examples of two-stage detectors are Faster R-CNN and Mask R-CNN.

RetinaNet is a one-stage object detector with focal loss as a classification. RetinaNet utilizes ResNet as its backbone. RetinaNet inherits the fast speed of previous one-stage detectors by avoiding the use of RPNs. Faster R-CNN extracts features from region proposals and then passes the region-of-interest (RoI) pooling layer to get the various size features as the input of the following classification and bounding-box regression fully-connected layers. Mask R-CNN [16] is an extending work to Faster R-CNN by using RoIAlign to extract a small feature map from each RoI and adding a parallel mask branch. The feature pyramid network (FPN) is the backbone that extracts RoI features from different levels of the feature pyramid according to extract features that achieve excellent accuracy and processing speeds. Given that higher-resolution feature maps are important for detecting small objects while lower-resolution feature maps are rich in semantic information, a feature pyramid network extracts significant features. 

**Figure 2:**
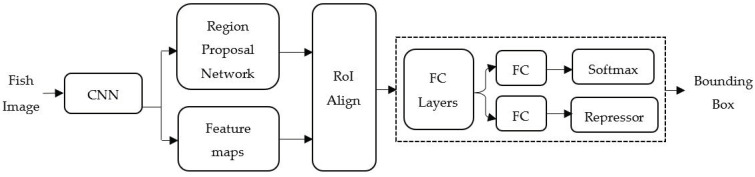
Mask R-CNN structure for Tilapia Detection.

Deep transfer learning comprises two steps: Firstly, the pre-training step and secondly, the post-training step. The pre-training step loads the learned weights from the pre-trained model as initial values for the deep learning network. For the post-training step, the deep learning network will learn and fine-tune the weight given by the Tilapia-image dataset. Deep transfer learning has the advantage of reducing learning time and increasing the accuracy of the model. The COCO-pre-trained Mask region R-CNN model was employed to determine the initial value of the deep learning architecture. Mask R-CNN is an object detection algorithm that performs target detection, target classification, and instance segmentation simultaneously in a neural network. Mask R-CNN returns two outputs that are a class and a bounding-box offset, as illustrated in Figure 2, where FC depicts fully-connected layers. A *m* × *m* mask representation encodes the spatial structure from an input image by the pixel-to-pixel method that corresponds to the convolutions. The *m* × *m* mask is generated from a region of interest (RoI) by using a fully convolutional network (FCN) with a per-pixel sigmoid and a binary loss to semantic segmentation. This naturally leads Mask R-CNN to maintain the 2-dimentinal spatial layout rather than transform it into a vector representation. 

Mask R-CNN consists of two components. Firstly, the backbone network of the proposed method is based on ResNet. ResNet consists of many stacks of residual units. Each unit can be expressed as in Equation (1), where $x_{l}$ and $x_{L}$ indicate an input feature to the *l*th Residual Unit and an output of any deeper unit *L* [24]:

(1) $$x_{L}=x_{l}+\sum_{i=l}^{L-1}F\left(x_{i},W_{i}\right)$$

where F(·) is a residual function and $\overset{L-1}{\underset{i=l}{\sum }}F\left(\cdot \right)$ is a residual function. The $W_{i}=W_{i,y|1\leq y\leq Layer}$ term is a set of weights (and biases) associated with the *l*th Residual Unit. A 3 × 3 convolution layer has been set for RPN. Secondly, RoIAlign performs per-pixel preservation of spatial features extraction by using a fully convolutional network and RoIPool for the feature map. Mask R-CNN applies a multi-loss function during the learning to evaluate the model and ensure its fitting to unseen data. This loss function is computed as a weighted total sum of various losses during the training at every phase of the model on each proposal RoI, which is shown by Equation (2). This weighted loss is defined as [25]:

(2) $$Loss=L_{class}+L_{BB}+L_{mask}$$

where $L_{class}$, $L_{BB}$, and $L_{mask}$ represent the classification loss, bounding-box loss, and the average binary cross-entropy loss, respectively. The $L_{class}$ shows the convergence of the predictions to the true class. $L_{class}$ combines the classification loss during the training of RPN and Mask R-CNN heads. $L_{BB}$ shows how well the model localizes objects and it combines the bounding-box localization loss during the training of RPN and Mask R-CNN heads. The $L_{class}$ and $L_{BB}$ losses are computed by Equations (3) and (4):

(3) $$L_{class}\left(p,u\right)=-\log p_{u}$$

where $L_{class}\left(p,u\right)$ is the predicted probability of ground truth class u for each positive bounding box.

(4) $$L_{BB}\left(t^{u},v\right)=\sum_{i\epsilon \left\{x,y,w,h\right\}}\left[L_{1}^{smooth}\left(t_{i}^{u}-v_{i}\right)\right]$$

where $L_{1}^{smooth}\left(x\right)=\{\begin{array}{cc} 0.5x^{2} & if\left|x\right|<1\\ \left|x\right|-0.5 & otherwise \end{array}$ and $L_{1}^{smooth}\left(t_{i}^{u}-v_{i}\right)$ are the predicted bounding-box for class u and ground truth bounding-box *v* for each input i.

The $L_{mask}$ has K×m×m dimensional output for each RoI where *K* represents a number of a class and m×m is a matrix representation of the class. A per-pixel sigmoid is applied and the $L_{mask}$ is computed using the average binary cross-entropy loss that the *K* mask is associated with the *K*th class, i.e., K=1=Tilapia. The $L_{mask}$ can be expressed in Equation (5) [26,27]:

(5) $$L_{mask}=\frac{1}{m^{2}}\sum_{i=1}^{m*m}\left(\log P_{i,j}^{K}\right)$$

where $P_{i,j}^{K}$ denotes the *i*th pixel of the *j*th generated mask. The backbone network has used a 101-layer ResNet and a 3 × 3 convolution layer has been set for RPN. Secondly, RoIAlign performs a per-pixel preservation of spatial features extraction by using a fully convolutional network and RoIPool for the feature map. This network outputs a *K* × *m* × *m* mask representation that is upscaled and the channels are reduced to 256 using a *m* × *m* convolution, where *K* is the number of classes, i.e., *K* = 1, and *m* = 28 for the ResNet_101 network as a backbone. All training parameters use the same values, where the batch size is 128 images, the learning rate is 2.5 × 10^−4^, and the maximum iterations are 300.

The TDet model delivers the bounding-box output as a set of coordinate points (x, y) of a detected fish. The coordinate points from the bounding box were extracted to compute the length and width of the detected fish. However, these measurements are subject to perspective projection (pixel units). The fish size in perspective projection relies on the depth between the fish and the camera. This results in the fish body that is closer to the camera being wider and longer than those further away. Thus, the fish size due to perspective projection is essentially converted into real-measurement units of the fish’s actual size before estimating the weight of the Tilapia. 

### 2.2. Tilapia Weight Estimation

The next step is to estimate the weight that comprises three sub-steps: First, estimating the depth of the fish; second, converting the fish’s width and length from pixel to centimetre; and finally, determining the fish’s weight by using all estimated data of the fish by training the TDepE, TP2CME, and TWE models, respectively. These three models specifically required the following independent data and delivered the dependent output as shown in Table 1.

The three models are sequentially related to one another, where an output of the previous model is an input of the next model. The regression models of the TDepE (${\hat{y}}_{depth}$), TP2CME (${\hat{y}}_{l\_cm},\;{\hat{y}}_{w\_cm}$), and TWE (${\hat{y}}_{w}$) models can mathematically be expressed in Equations (6)–(8), respectively, as:

(6) $${\hat{y}}_{depth}=f\left(x_{age},x_{w\_pix},x_{h\_pix},a_{age},a_{w\_pix},a_{h\_pix}\right)+e_{depth}$$

(7) $${\hat{y}}_{l\_cm},\;{\hat{y}}_{w\_cm}=f\left(x_{age},x_{w\_pix},x_{h\_pix},{\hat{y}}_{depth},a_{age},a_{w\_pix},a_{h\_pix},a_{depth}\right)+e_{cm}$$

(8) $${\hat{y}}_{w}=f\left(x_{age},x_{w\_pix},x_{h\_pix},{\hat{y}}_{depth},{\hat{y}}_{l\_cm},\;{\hat{y}}_{w\_cm},a_{age},a_{w\_pix},a_{h\_pix},a_{depth},a_{w\_cm},a_{h\_cm}\right)+e_{w}$$

where $e_{depth}$, $e_{cm}$, and $e_{w}$ denotes an additive error term. The closed form equation to link all the above equations together is to be determined by the machine learning model. To achieve the goal, the regression models, i.e., Tilapia depth estimation, Tilapia pixel-to-centimetre estimation, and Tilapia weight estimation, were constructed by employing three well-known regression methods. The regression models are LR, RFR, and SVR. Linear regression is a linear model of relationship between independent variables and a dependent variable. The linear model is expressed in Equation (9):

(9) $$y=a_{0}+\sum_{j=1}^{J}a_{j}x_{j}\;$$

where $x_{j}$ and y denote the *j-*th independent variable and the dependent variable, respectively. The terms $\left\{a_{j},\;j=0,\;1,\ldots ,J\right\}$ are the coefficients of the model and *J* is the total number of features used for the regression. Secondly, random forest is a decision-tree extension by constructing a multitude of trees in a training period. Random forest is deep learning for classification or regression tasks. In the multitude trees, individual trees randomly select a subset of features. The optimal splitting point is determined by the predicted squared error as a criterion of a regression model. RFR output ($\hat{y}$) is based on a weighted sum of datapoints, as expressed in Equation (10):

(10) $$\hat{y}=\sum_{i=1}^{n}\left(\frac{1}{m}\sum_{j=1}^{m}W_{j}\left(x_{i},x^{\prime }\right)\right)y_{i}$$

where *x_i_* and *y_i_* denote the dataset and $w_{i}$ is a weight of *y_i_*. The *x*′ term represents the neighbour node that shares the same leaf in a tree *j* with the point *x_i_* [28]. The squared error is expressed in Equation (11):

(11) $$\min\sum_{i=1}^{n}{\left(y_{i}-w_{i}x_{i}\right)}^{2}$$

Finally, the support vector regression is an extension of the support vector machine for solving regression problems. The objective function of SVR is to minimize the coefficients by using the *l*_2_-norm of the coefficient vector [29,30] instead of the squared error, as expressed in Equation (12). The constraint called the maximum error (ϵ) is represented by the absolute error in Equation (13). The ϵ paremeter will be tuned by the regression function to gain the best fit line, where a hyperplane has a maximum number of points [31].

(12) $$\min\frac{1}{2}\|w^{2}\|$$

(13) $$s.t.\;\left|y_{i}-w_{i}x_{i}\right|\leq \epsilon$$

The ϵ value determines the distance of the support-vector line (so-called decision boundary) that deviates from the hyperplane line.

A subsequent training phase delivers the TDet, TDepE, TP2CME, and TWE models. The evaluation phase, as shown in Figure 1, will use these models for estimating the weight of the Tilapia given by an observed video input. An overview of the proposed Tilapia weight-estimation evaluation phase is explained in Algorithm 1. **Algorithm 1.** Overview of the Proposed Tilapia Weight-Estimation Evaluation Phase(1) Convert an observed video input to images:s[n]=s(nT)
(2) Enhance images in a case of turbid water:
  (2.1) Image sharpening by the convolution function $g_{1}\left(x,y\right)$:
$g_{1}\left(x,y\right)=\overset{a}{\underset{dx=-1}{\sum }}\overset{b}{\underset{dy=-b}{\sum }}\omega \left(dx,dy\right)s\left(x+dx,y+dy\right)$
where −a≤dx≤a and −b≤dy≤b, s(·) denotes the original image, and ω(·) is the filter kernel, i.e., sharpen, filter.
  (2.2) Color correction matrix (CCM) [32]:
$S={\left[S_{R}\;S_{G}\;S_{B}\;S_{W}\right]}^{T}$$\left[\begin{array}{c} C_{R}\\ C_{G}\\ C_{B} \end{array}\right]={\left(CCM.\left[\begin{array}{c} S_{R}\\ S_{G}\\ \begin{array}{c} S_{B}\\ S_{W} \end{array} \end{array}-\begin{array}{c} V_{offset}^{R}\\ V_{offset}^{G}\\ \begin{array}{c} V_{offset}^{B}\\ V_{offset}^{W} \end{array} \end{array}\right]\right)}^{1/\gamma }$
where $S_{R}\;S_{G}\;S_{B}\;S_{W}$ denote the red, green, blue, and white spaces; C is the color-component vector; and $V_{offset}$ is the offset vector.
  (2.3) Exposure adjustment $g_{2}\left(x,y\right)$:
$g_{2}\left(x,y\right)=\alpha \cdot s\left(x,y\right)+\beta$
where α>0 is the gain and β represents the bias parameter.

(3) Detect the length and width of Tilapia:
${\hat{x}}_{w\_pix},{\hat{x}}_{h\_pix}=Mask\;R-CNN_{BB}\left(g_{2}\left(x,y\right)\right)$
(4) Estimate the depth of each detected Tilapia:
${\hat{y}}_{depth}=f\left(x_{age},{\hat{x}}_{w\_pix},{\hat{x}}_{h\_pix},a_{age},a_{w\_pix},a_{h\_pix}\right)+e_{depth}$
(5) Convert Tilapia size from pixel to centimeter:
${\hat{y}}_{l\_cm},\;{\hat{y}}_{w\_cm}=f\left(x_{age},x_{w\_pix},x_{h\_pix},{\hat{y}}_{depth},a_{age},a_{w\_pix},a_{h\_pix},a_{depth}\right)+e_{cm}$
(6) Estimate the weight of individual detected Tilapia.
${\hat{y}}_{w}=f\left(x_{age},x_{w\_pix},x_{h\_pix},{\hat{y}}_{depth},{\hat{y}}_{l\_cm},\;{\hat{y}}_{w\_cm},a_{age},a_{w\_pix},a_{h\_pix},a_{depth},a_{w\_cm},a_{h\_cm}\right)+e_{w}$

## 3. Experimental Results and Analysis

### 3.1. Data Collection

The Tilapia were raised in 3 tanks where each tank contained 30 fish. The tanks are round with a radius of 1.5 m and a depth of 1.8 m. A new fish cultivation method was used for the efficient feeding of fish, which is called the biofloc culture. The biofloc tank is a microorganism cultured fish, thus the biofloc microorganisms caused the water to turn turbid. Bacteria are put into an aquaculture system to convert nitrogen from the water into protein. The protein will be the food of the fish. The wastewater that contains nitrates, nitrites, and ammonia will be treated and reused as supermolecule feed. Biofloc fish feeding is a technology that feeds aquaculture systems with macroaggregates that decrease the fish diet cost and improve the aquatic environment of a fish tank.

**Figure 3:**
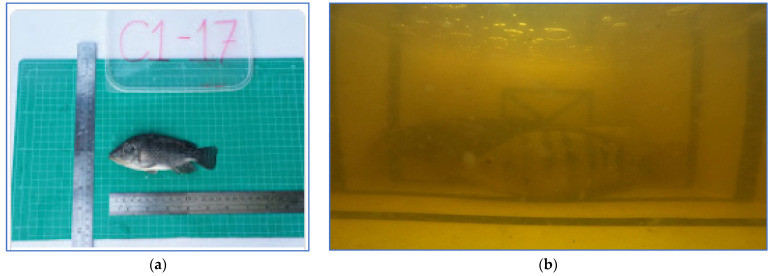
Example of Tilapia images from (**a**) studio; (**b**) biofloc tank.

Datasets developed in this research can be categorized into the (a) Tilapia-image datasets and (b) Tilapia-file datasets. Firstly, the Tilapia-image datasets are in-house curated from two sources: studio-based photography of the off tanks and from video recordings of the tanks, as shown in Figure 3.

The studio-based photography is set up by using a camera (Cannon EOS 200D II) mounted in a fixed position that is 0.5 m from the fish and parallel to the platform with a resolution of 1920 × 1080 pixels. The fish were weighed with an electronic scale before photographing. The video recording (GoPro Hero 8 and waterproof case) was carried out by sampling five fish from the tanks and putting them into the recording tanks. The videos were recorded at a resolution of 1920 × 1080 pixels, with a frame rate of 60 fps and 8-bit RGB. Data collection of each fish from the studio and video was performed, including age (weeks), width and length throughout the fish in centimeters (cm), and the weight of the fish in grams. Secondly, the Tilapia-file dataset was created for training the regression models. The Tilapia-file dataset includes three attributes, which are the fish’s age, the physical dimensions of the fish in pixel and centimetre units, and the depth between the fish and the camera. The two Tilapia datasets were employed for training the models to estimate the Tilapia’s weight. 

### 3.2. Data Preparation

**Figure 4:**
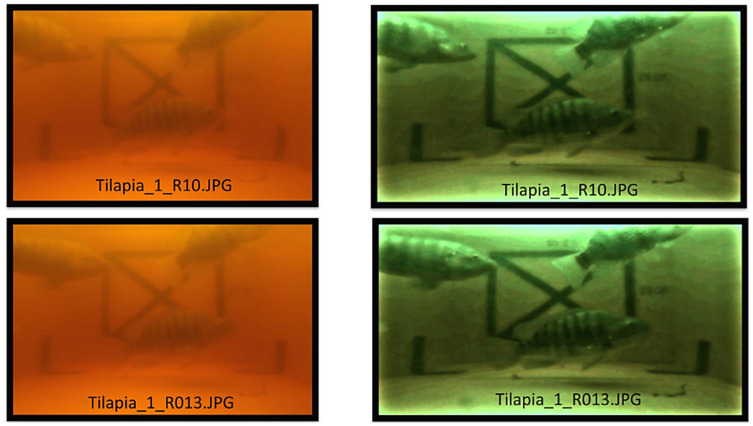
Comparison between original images (**left**) and enhanced images (**right**).

Data pre-processing of the videos refers to the proposed processes of converting video to images, an image enhancing process for the biofloc tanks, and an image annotation process. All fish images have 24-bits of a red, green, and blue channel and each channel has 256 intensity levels. Both images from the studio and videos are required in the annotation process. In the case of videos, firstly, the video-to-image process is the diminution of a continuous-time signal *s*(*t*) to a discrete-time signal. The original signal will be sampled at a *T* period to obtain a series of discreate signals that instantaneously become the original continuous signal. The sampling image process can be expressed in (14) as:

(14) s[n]=s(nT)

where *n* denotes the sequence index of the *T* period. The biofloc tank is a microorganism cultured fish, thus biofloc microorganisms cause the water to turn turbid. Therefore, the sampled images of the biofloc tanks were pre-processed and enhanced in order to be able to identify fish by applying the image enhancement process. The image enhancement process consists of four steps, i.e., image sharpen, color filter, color balance, and exposure adjustment, where the values of the individual channels of an image are modified to improve the images’ quality. Starting with image sharpening, this involves increasing the contrast, edge detection, noise suppression, and Gaussian Blur algorithms [33,34]. Next, color filter and color balance aim to adjust the color temperature by using curve shifting [35]. Color balance is used to manipulate any unwanted color that dominates an image by estimating the illumination and applying correction to the image [36]. Finally, exposure adjustment is focused on controlling the light of on an image via two parameters: the exposure time and the light sensitivity of the image [37]. Enhanced images are presented in Figure 4.

**Figure 5:**
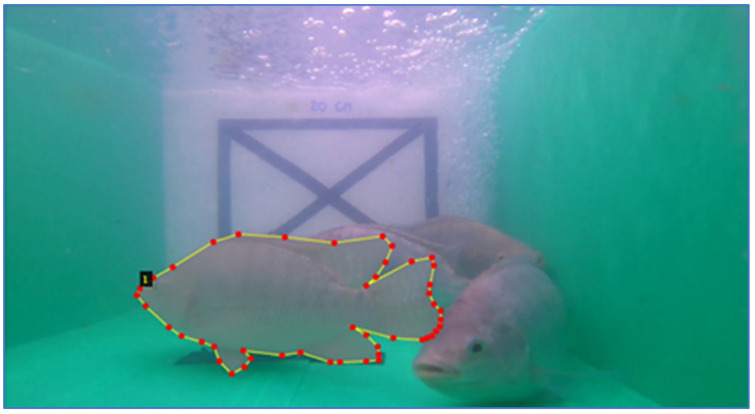
A coordinate-position set of a fish via the image annotation process.

The image annotation is the process of describing the target objects in an image, as shown in Figure 5. The descriptive data allow the computer to interpret the image in a similar way as human understanding. A computer understands digital images by extracting data from a real-world image into numerical information then interprets the information via a deep learning algorithm. Visual images will be provided as description data of a target object in the image, which is known as image annotation. In a similar way to a human learning an object, image annotation is the procedure of labeling images to train a deep learning model. The deep learning algorithm then transforms the image by disentangling symbolic information into numerical sparse information through the convolution process. Finally, an objective model is then learned by using the fully-connected MLP networks given by the information from the convolution phase. Three attributes were defined for the explanation of a fish, which are age (weeks); distance between a fish and a camera, i.e., so-called depth (cm); and a coordinate-position set of a fish. The fish annotation yields a JSON file as an output of the process. This process is performed via the Visual Geometry Group Image Annotator website (https://www.robots.ox.ac.uk/~vgg/software/via/via_demo.html accessed on 20 June 2022).

The experimental scheme has been established for 3 months, where the start age of the Tilapia was 20 weeks. The assumptions made in the work are that the Tilapia weight can be estimated with a good level of accuracy. The input of the proposed TWE-DRL algorithm, as illustrated in Figure 2, is made up of two types, where images (i.e., studio) and video signals are processed five times every two weeks. The studio-based photography is set up by using a camera mounted in a fixed position that is 0.5 m from the fish and is parallel to the platform, with a resolution of 1920 × 1080 pixels. The Tilapias were recorded in a turbid water recording tank (i.e., in video) with a resolution of 1920 × 1080 pixels, with a frame rate of 60 fps and 8-bit RGB. For the first actual weighting of 20-week-old Tilapia, the average weight was 166.45 ± 26.38 g, while it was 482.24 ± 91.64 g at the last weighing for 28-week-old Tilapia. The Tilapia-image dataset contains 5037 images, where 750 images were from studio and 4287 images were from video, while Tilapia-file dataset contains 2777 files. The video recording will be converted to images by every second and then the quality of the images will be improved by the image enhancement process. Next, the enhanced images will be used as input data for the Tilapia detection step, which is based on deep transfer learning. All one-class training parameters use the same values, where the backbone is a RestNet learning network, the batch size is 128 images, the learning rate is 2.5 × 10^−4^, and the maximum iterations are 300. The output of the detection step will be the input of the Tilapia weight-estimation step that is based on regression models. The regression models are LR, RFR with 2 level maximum depth, and SVR with radial basis function (RBF) methods. The inputs of individual TDepE, TP2CME, and TWE are expressed in Table 1 and Equations (6)–(8). Finally, the proposed methods will deliver the estimated weight of Tilapia in a data file. 

The experimental results have been conducted in two major sections: The first section rigorously determines the optimal models of Tilapia detection, i.e., TDet, and Tilapia weight estimation, i.e., TDepE, TP2CME, and TWE. The second section verifies the effectiveness of the proposed Tilapia weight-estimation methods. The Tilapia-images dataset has 4287 images with various ages, which were split into 60% for training and the rest for testing. The Tilapia-file dataset contains 2777 files, which were partitioned into 70% for training and the rest for testing. The number of training and testing data corresponding to each model is presented in Table 2.

The proposed TWE algorithm is used to train the various regression models and its effectiveness is assessed using the following measurements in Equations (15) and (16):

The mean absolute error (MAE):

(15) $$\mathrm{MAE}\left(y,\hat{y}\right)=\frac{1}{n_{samples}}\sum_{i=0}^{n_{samples}-1}\left|y_{i}-{\hat{y}}_{i}\right|$$

The coefficient of determination, *R*^2^:

(16) $$R^{2}\left(y,\hat{y}\right)=1-\frac{\sum_{i=1}^{n}{\left(y_{i}-{\hat{y}}_{i}\right)}^{2}}{\sum_{i=1}^{n}{\left(y_{i}-\bar{y}\right)}^{2}}$$

The experiments were conducted using the following hardware and software environments: hardware environment employed the AMD Ryzen 9 4900H with Radeon Graphics 3.30 GHz, Nvidia GeForce GTX 1660 Ti, 16.00 GB DDR4. Software tools are Python 3.x and TensorFlow-GPU v2.3.0, Keras v2.4.3 in Windows 10 operating system.

### 3.3. Determining the Optimal Tilapia Detection Models 

The state-of-the-art deep learning networks have been used to determine the optimal Tilapia detecting models of Mask R-CNN, Faster R-CNN, RetinaNet, and YOLO. YOLOv5 has been used as Tilapia detection experiment, where the following parameters have been determined: scaled weight decay at 0.0005, training for 300 epochs, batch size at 128, and a learning rate of 0.01, as well, the optimizer that is relied on is a Gradient descent with momentum optimizer. All training parameters used the same values, where the batch size was 128 images, the learning rate was 2.5 × 10^−4^, and the maximum iterations are 300.

The object-detecting performance of the three methods were averaged over multiple Intersection-over-Union (IoU) scores, called AP, which used 10 IoU with various thresholds. The experimental results are shown in Table 3.

**Figure 6:**
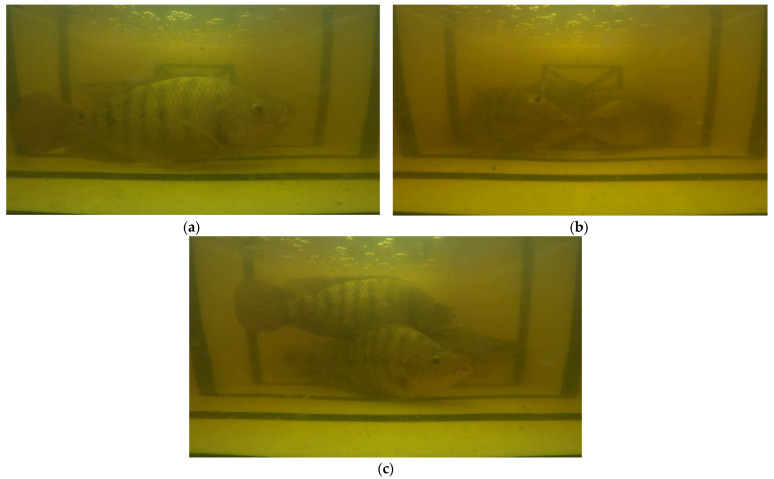
Sample of observed Tilapia in turbid water where (**a**) a single Tilapia; (**b**) two Tilapia with more than 50% of a body size appearance; (**c**) multiple Tilapia overlapping.

**Figure 7:**
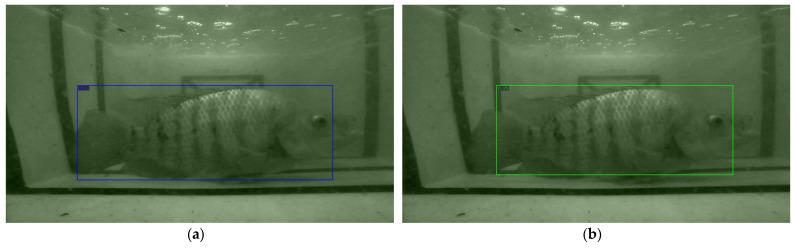

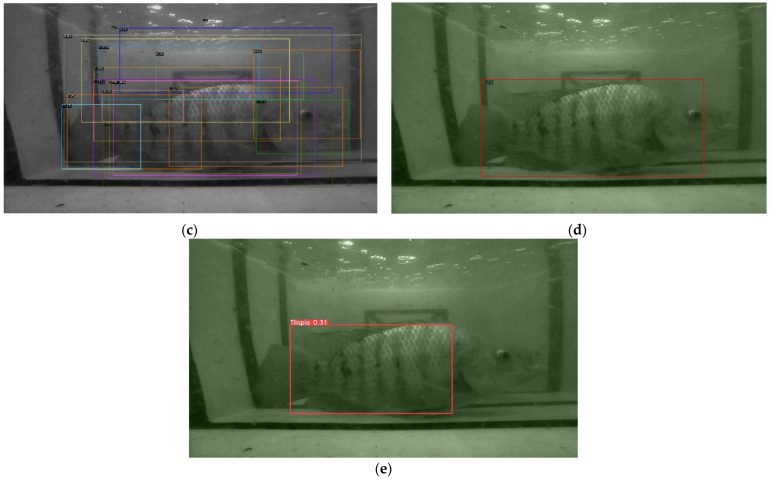
Sample of a single Tilapia. (**a**) Faster R-CNN: model detected a Tilapia with 0.87 probability score; (**b**) Mask R-CNN: model detected a Tilapia with 0.95 probability score; (**c**,**d**) RetinaNet: model drew 19 bounding boxes with the highest, average, and standard deviation of probability scores at 0.73, 0.11, and 0.15, respectively; (**e**) YOLO: model detected a Tilapia with 0.31 probability score. YOLO can only detect a sample of a single Tilapia underwater in (**a**) but unsuccessful in scenarios (**b**,**c**).

**Figure 8:**
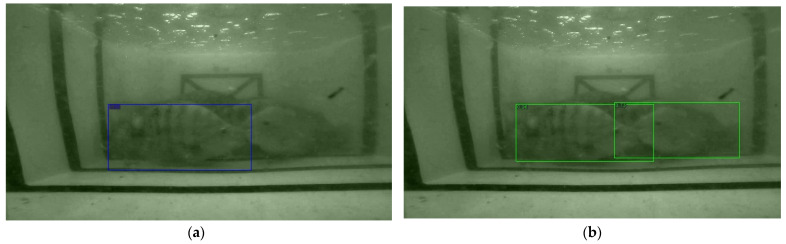

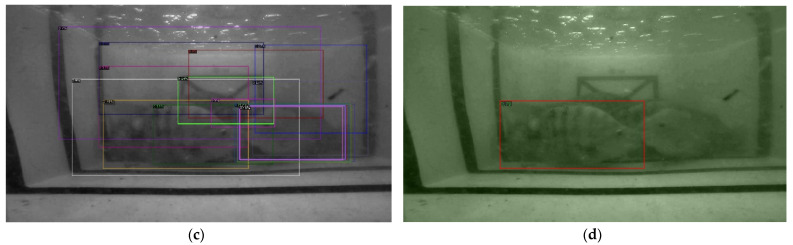
Sample of two Tilapia with more than 50% of a body size appearance. (**a**) Faster R-CNN: model detected a Tilapia with 0.88 probability score; (**b**) Mask R-CNN: model detected a Tilapia with 0.96 and 0.73 probability scores from left to right; (**c**,**d**) RetinaNet: model drew 28 bounding boxes with the highest, average, and standard deviation of probability scores at 0.79, 0.13, and 0.15, respectively.

**Figure 9:**
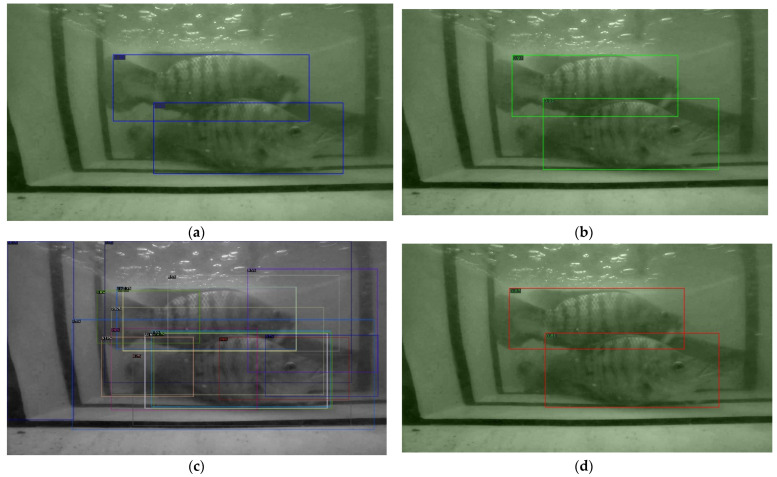
Sample of multiple Tilapia overlapping. (**a**) Faster R-CNN: model detected a Tilapia with 0.86 and 0.85 probability scores from left to right; (**b**) Mask R-CNN: model detected a Tilapia with 0.97 and 0.92 probability scores from left to right; (**c**,**d**) RetinaNet: model drew 19 bounding boxes with the highest, average, and standard deviation of probability scores at 0.83, 0.17, and 0.25, respectively.

The detection results of the above detection networks are presented in three scenarios, which are a single Tilapia, two Tilapia with more than 50% of a body size appearance, and multiple Tilapia overlapping. The samples of the observed images from the three scenarios are shown in Figure 6, and the detected results are then illustrated in Figure 7, Figure 8 and Figure 9.

The results from Figure 7, Figure 8 and Figure 9 have shown that Mask R-CNN yields the highest AP scores among the three thresholds. The reason is due to the RoIAlign operation of Mask R-CNN, which is able to extract features from small objects, i.e., Tilapia in blurred, low light, and noisy backgrounds. This leads to a higher accuracy than the Faster R-CNN and RetinaNet models. Therefore, TDet is built based on the Mask R-CNN model for determining the length and width of Tilapia from images. The TDet model obtained by the YOLOv5 framework is able to detect the case of a single Tilapia. In the cases with more complex scenarios where the fish appear to be blurry and small, as in Figure 6b, or chaotic, as in Figure 6c, the YOLOv5 model is unable to detect the fish. On the other hand, Mask R-CNN outperformed TDet-based YOLOv5 for the complex scenarios. YOLO network architecture employs convolutional neural networks (CNN) for extracting the significant features of the fish. A regression problem is treated by a single forward propagation to provide the class probabilities of the detected Tilapia. Therefore, it is difficult for YOLOv5 to extract key features from intricate images due to the spatial plane coordinate, as the grid location constrains the algorithm. Mask R-CNN takes advantage of RoI and RoIAlign processes for selecting the high-level features. This leads to a higher accuracy than all the other comparison methods.

### 3.4. Determining the Regression Learning Methods for the TDepE, TP2CME, and TWE Models

The Tilapia-file dataset was used for training the TDepE, TP2CME, and TWE models by splitting 80% of data is for training and the remaining data is for testing. The three sub-steps of Tilapia weight estimation are sequentially performed. A grid search and validation dataset were used to find the optimal parameter of the TDepE, TP2CME, and TWE models by specifying every combination of the parameter settings. Grid search passes all combinations of the hyperparameters one-by-one into the model to determine the optimal values for a given model. Hyperparameters are the variables that are used to evaluate the optimal parameters of the model. The hyperparameters for RFR and SVR were determined, which are the {maximum depth, maximum features, minimum samples leaf, minimum sample split, the number of estimators} and {regularization parameter, kernel coefficient, kernel types} sets, respectively. Finally, grid search delivers the set of hyperparameters that gives the best performance for the model. The validation dataset is used to determine the hyperparameters of each of the machine learning models in TDepE, TP2CME, and TWE. Next, TDepE model is firstly presented with the chosen regression method and then followed by the rest of the steps in succession.

#### 3.4.1. Tilapia Depth Estimation Performance

**Figure 10:**
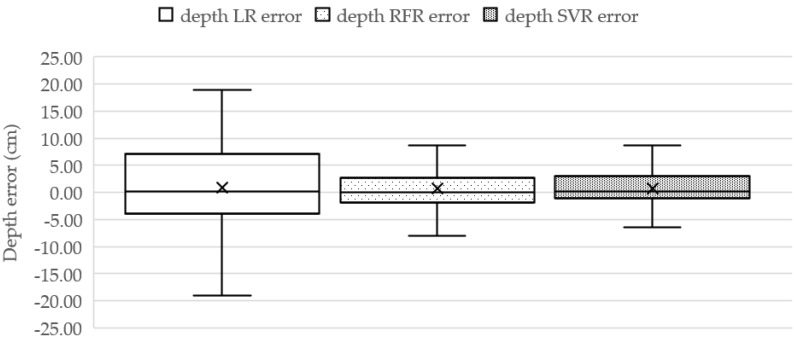
Box−plot comparison of Tilapia Depth Estimation error of LR, RFR, and SVR methods.

The TDepE model was trained by learning data consisting of the age, the length, and the width of the fish (pixel), as well as the actual depth of the fish. In terms of the performance, the obtained TDepE models based on LR, RFR with 2 level maximum depth, and SVR with radial basis function (RBF) methods [38,39] are illustrated in Table 2 and Figure 10, respectively. The RBF kernel [40] is expressed in Equation (17) as:

(17) $$K\left(X_{1},X_{2}\right)=exp(-\frac{{\left\|X_{1}-X_{2}\right\|}^{2}}{2\sigma^{2}})$$

where $\sigma^{2}$ denotes the variance as the hyperparameter and $\left\|X_{1}-X_{2}\right\|$ represents the Euclidean (*L₂*-norm) Distance between two points *X*_1_ and *X*_2_. The distance between the fish and the camera is between 5 cm and 60 cm. Depth data of Tilapia-file dataset was collected by using a manual visual distance estimation method with reference to distance markers every 10 cm, which were installed in the fish recording cube. The depth estimating performance of the LR, RFR, and SVR models are explicitly presented in Figure 10, and the actual depth values are widely spread from 5 cm to 50 cm with a 23.13 average depth and a 15.77 standard deviation (S.D.) score. The TDepE model-based SVR can estimate the depth close to the actual depth distribution.

The depth estimating performance is evaluated by measuring MAE along with the average errors and S.D. values in Table 3. The SVR model provides the best scores for MAE, *R*^2^, and the MAE ratio over the LR and RFR models at 5.52 cm and 1.56 cm for the MAE values, 0.46 and 0.12 for the *R*^2^ values, and 18.67 and 2.82 for the MAE ratio values, respectively.

According to Table 4, the SVR method yields outstanding performance for estimating depth of the fish. Therefore, the TDepE-based SVR model is set for the depth estimation step. Next, the experiment aims to figure out the regression method for TP2CME and TWE by measuring weight-estimating accuracy.

#### 3.4.2. Tilapia Pixel-to-Centimeter Estimation and Tilapia Weight Performance

The three investigational cases were set as presented in Table 4 for TP2CME and TWE. Each case starts from the TDet and TDepE steps. The TP2CME model learned from the fish attributes, including age, length and width of the fish in pixel units, and depth of the fish. The TWE model requires the length and the width of the fish in cm units. The experimental cases consist of two steps of TP2CME and TWE. The TP2CME for the individual case used a different regression learning method. Hence, we have three main cases of SVR, RFR, and LR, where the depth estimation is based on SVR—as shown in Table 5. Finally, the TWE step of the three cases is then applied for all three regression methods to estimate the weight of the fish.

**Figure 11:**
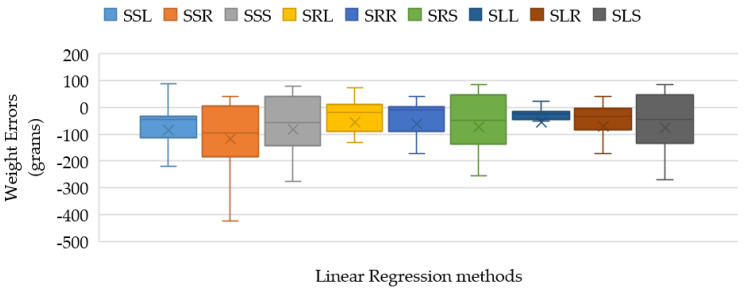
Box-plot comparison of Tilapia-weight estimating errors of the nine candidates corresponding to Case 1, Case 2, and Case 3 for determining the regression method to TP2CME and TWE.

The box plots represent the weight-estimation errors of the three cases, as illustrated in Figure 11. The TP2CME- and TWE-based LR models yield the minimum errors and deviation that are obviously noticed by the smallest size of the weight-error box from SLL with the average error at 43.80 ± 47.69 g.

The MAE and *R*^2^ scores for all cases are presented in Table 6. The SLL method yields the best estimating performance among all cases with the MAE, R2, and MAE ratio values at 42.54 cm, 0.70, and 60.77, respectively.

According to Table 6, the weight-estimating procedure can be recapped by the regression-learning solution of the TDepE, TP2CME, and TWE steps, which are the SVR model, the LR model, and the LR model, respectively.

**Figure 12:**
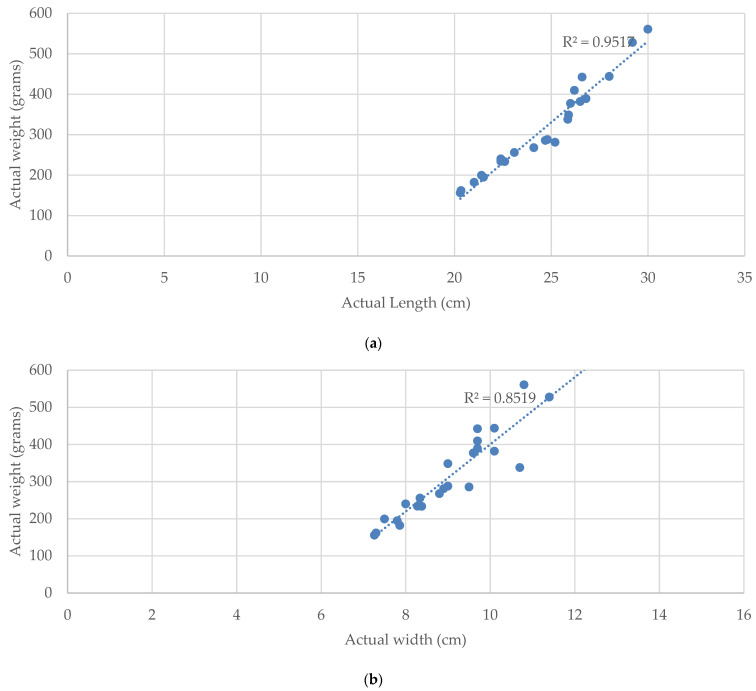
*R*^2^ scores of LR regression on relationship of actual weight with (**a**) actual length; (**b**) actual width.

The relationship of the weight and size of Tilapia with linear regression by the *R*^2^ measurement is shown in Figure 12. The *R*^2^ value of LR is 0.95 for the weight–length relationship and 0.85 for the weight–width relationship, respectively. This result shows that the length and width of Tilapia is significantly correlated to the weight of Tilapia.

According to Figure 12, the *R*^2^ values indicate the strength of the relationship between the proposed TWE-DRL model and the dependent length and width variable at 95.17% and 85.19%, respectively.

### 3.5. Tilapia Weight Estimation Performance

This section demonstrates the weight estimation performance of the proposed TWE-DRL method against the benchmarks of seven fish weight estimation-based areas (A) of the fish’s size in [6]. The area-based weight estimation methods with various coefficients can be expressed through the following equations in Equations (18)–(24).

Power based: W_1_ = 1.70A^3/2^ (18)

Power based: W_2_ = 0.124A^1.55^ (19)

Exponential: W_3_ = 75.505e^0.008A^ (20)

Linear: W_4_= 2.6609A − 141.14 (21)

Logarithmic: W_5_ = 448.84ln(A) − 1984.1 (22)

Polynomial: W_6_ = 0.0048A^2^ + 0.9309A + 7.8245 (23)

**Figure 13:**
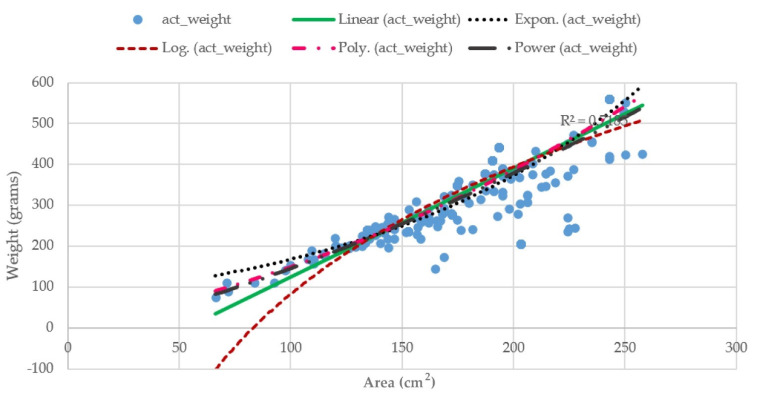
Cartesian coordinates of a point of the Euclidean plane for determining the coefficients of Equations (14)–(18).

Power: W_7_ = 0.2501A^1.3821^ (24)

where an area (A) of the fish’s body in cm^2^ have been computed from multiplying the length and the width of that fish, which was obtained from the Tilapia detection phase with a coefficient, i.e., A = length × width × coefficient. The coefficients in Equations (20)–(24) were obtained by formulating lines corresponding to individual equations for representing the relationship between the actual fish’s area and its actual weight. The plots are illustrated in Figure 13.

The evaluated Tilapia datasets were established for 3 months and recorded every 2 weeks, with the Tilapia being 20-week-olds. All comparison methods were provided by the estimated length and width of the Tilapia that were obtained from the TDet and TP2CME models of the proposed method. The estimated weight results are presented in Table 7.

According to the results in Table 7, the proposed methods obtained the smallest MAE score and highest R^2^ scores, where an average error is 42.54 g from the actual weight of fish. The regression models of the proposed methods can predict that the weight of the Tilapia has a 70% fit to the actual weight. The proposed method estimates the fish weight from the length and width of the fish, while the other methods use the area of the fish. From Figure 9, the R^2^ values of length and width are 0.9517 and 0.8519, while the maximum R^2^ value from Equations (14)–(18) is 0.7507. Hence, the length and width of the fish is significantly accurate for estimating the weight of the fish. Therefore, the proposed TWE-DRL method yields the highest accuracy over the area-based weight-estimation methods.

**Figure 14:**
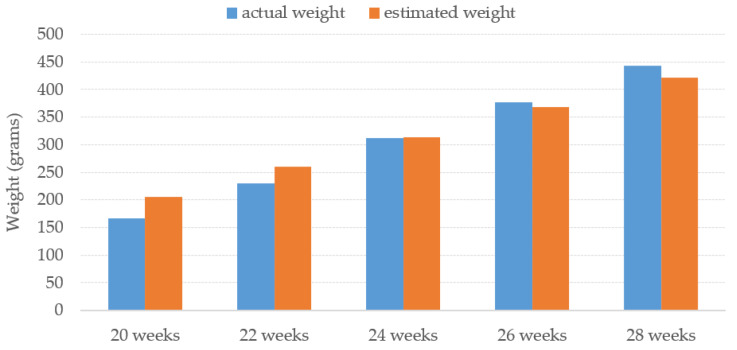
Proposed TWE-DRL performance presents average estimated weight of Tilapia with various age in turbid water.

The average estimated weight of the proposed method for each week is illustrated in Figure 14 against the average actual weight of the Tilapia. The results of Tilapia weight estimation from turbid water by the proposed TWE-DRL method vary by the fish’s age and are plotted compared to the actual weight. The proposed TWE-DRL method has estimated the Tilapia weights consistently and is tallied with the actual Tilapia weight patterns by using the TDet, TDepE, TP2CME, and TWE models. The obtained results show that across the eight weeks, the proposed method has only accrued an estimated weight error of 30.30 (±23.09) grams. The proposed approach can perform at high accuracies and is able to track the weight evolution of the fish in the tank from week to week. In addition, once the system has completed the estimation processes, all the estimated results will be saved to a Microsoft Excel file as an output of the system.

**Figure 15:**
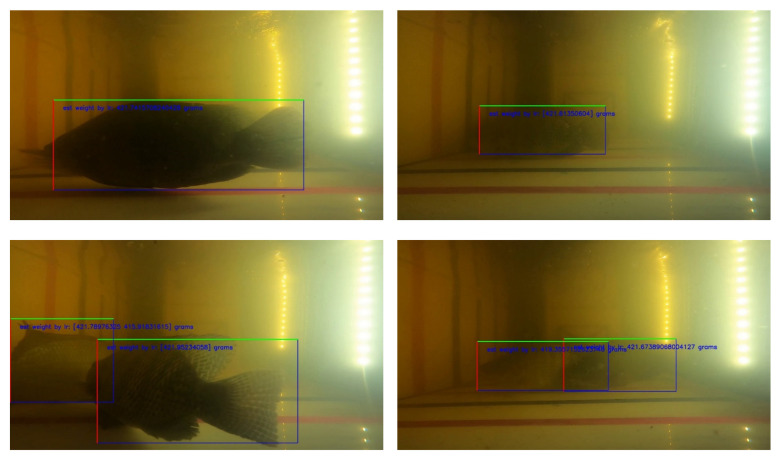
Examples of Tilapia Weight-Estimation Results in turbid water for two cases that are near and far from camera: (1) the near camera: a single fish and two overlapping (**top** and **down left**) and (2) the far from camera: a single fish and two overlapping (**top** and **down right**).

Examples of the fish body and size detection results are shown in Figure 15, where fish were recorded from underwater at various depths. The TDet model can detect multiple fish in the image with their bodies aligned horizontally in the image. The proposed method can precisely detect the body size of each fish even when the fish overlap, as presented in Figure 15.

**Figure 16:**
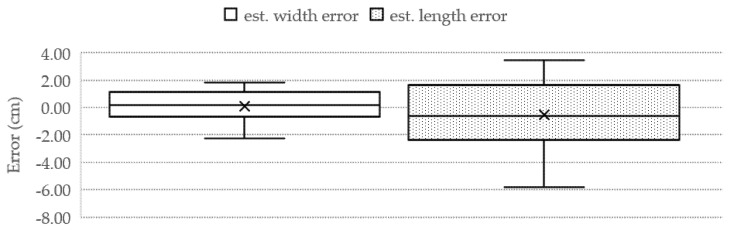
Tilapia Detection performance presented via box−plot estimated errors of length (**right**) and width (**left**) of Tilapia’s size.

**Figure 17:**
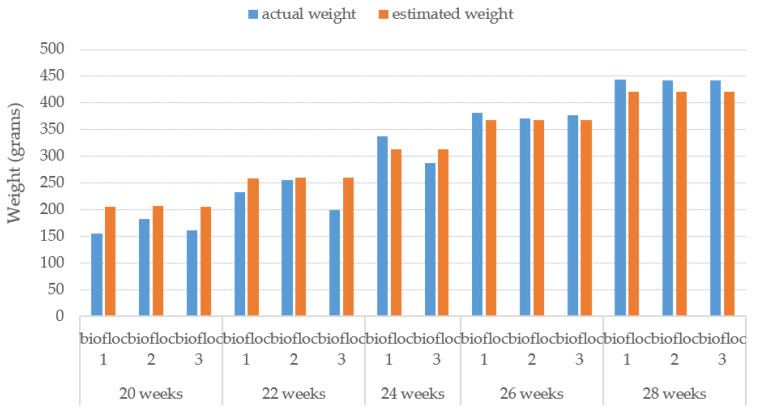
Comparison of the distribution of actual weight and estimated weight by proposed TWE-DRL method.

The proposed TWE-DRL method can detect fish in turbid water in a variety of distances, both near and far from the camera recorder. The proposed algorithm for the TDet results is set at 0.8 for the probability criterion so that images with a probability equal to or greater than 0.8 will be passed through for further processing. Subsequently, the size of the fish in pixels was converted to cm with the TP2CME model using the fish size data from the detecting process together with the depth information obtained from the TDepE model. Turbid water and the depth of the fish have a major influence on fish detection—for example, two fish that overlap with one another at a further distance from the camera. The performance of the Tilapia size estimation from the proposed TWE-DRL method is shown by MAE, while the box plot values are shown in Figure 16. The estimated error accrued by the proposed method is 2.3 cm and 0.96 cm for length and width, respectively. The actual fish have a length and width that range from 20–30 cm and 7–12 cm, depending on the age of the fish. The estimated-length error of the fish, as shown in Figure 16, has a wider spread error than the estimated-width error. This is caused by a wider range of the fish’s actual length than that of the fish’s width. This leads to the consistency for estimating the performance of the proposed TWE method. In some cases, the proposed TWE method may detect the overlapping fish as a single fish. The Tilapia was raised in 3 biofloc tanks for 3 months, and the Tilapia were 20 weeks old at the start. The Tilapia were recorded underwater every two weeks. The estimated weight of the Tilapia from 20-weeks-old to 28-weeks-old are plotted against their actual weight from the video, which is related to the actual length of the Tilapia, as illustrated in Figure 17.

Note that, at 24 weeks of age, the second tank has no data due to all the fish dying and a new set of fish from a reserve tank was supplied instead. The proposed TWE-DRL method has estimated the Tilapia weight given by observed videos where the results show a close resemblance to the actual weight. This is to show the correctness of the proposed method.

**Figure 18:**
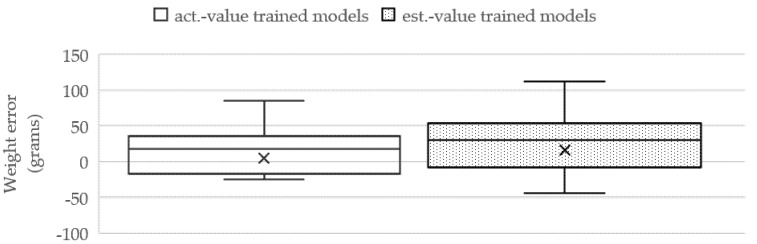
Comparison of the estimated weight of the proposed TWE method between the actual −value trained models and the estimated-value trained models.

The next section will demonstrate the performance of the proposed TWE-DRL method, which is given by a dataset of estimates derived from the models. All attributes in the estimated-value dataset were obtained by the models proposed in this paper, i.e., TDepE, TP2CME, and TWE. This dataset was used to train the TDepE, TP2CME, and TWE models by following the same steps in Section 3.4.1 and Section 3.4.2. From the experiments, it was found that the SVR, RFR, and LR methods of the TDepE, TP2CME, and TWE models yield the best estimation results. The fish weights predicted from the estimated-value models were compared with the weight results obtained from the actual-value models. This is shown in Figure 18. The estimated weight using the trained models performs with a slightly higher error than the actual value trained model with 14.50 cm of MAE across the test dataset.

The well-known weight estimation of fish can be categorized into two cases, in case of off-water and underwater scenarios. Firstly, in the case of off-water, fish weight-estimation-based CNNs are proposed in Refs. [5,41] by using ResNet-34 and LinkNet-34 for segmenting fish images, then the weight of the fish is computed from the surface area of the fish. The datasets from this research contain 2445 images of fish with weights in the range of 15 g to 2500 g, where the distance between the fish and the camera is constant in all images. Thus, the depth of the fish will be provided as a priori information. The mass estimation performance of Ref. [42] yields the R^2^ value of 0.976. Another off-tank method is presented in Ref. [5], the dataset contains 694 images of fish from the 22 species of fish from 9 tributaries where images were captured. The fish’s weight is between 500 g and 1200 g. Six cameras were set at a fixed distance, with three being near-infrared cameras and three being general cameras. The output of the DCNNs phase is passed into the regression phase where the final output will be an averaged value of nine images. The performance of the weight estimation from Ref. [5] gains an MAE of 634 g. Secondly, underwater fish-weight estimation is presented in Ref. [7], where the fish weight-estimation methods are the weight prediction system for Nile Tilapia. This method uses stereo cameras for distance measurements and captured 10 Tilapia in a tank of clear water for 3 weeks. The fish’s weight is in a range of 24 g to 41 g. CNNs are used for fish detection. Regression equations are proposed for computing the depth of the fish, converting pixel-to-cm, and weight prediction. The correlation of the weight and length based on linear regression has an R^2^ value of 0.87. The fish’s weight from the proposed TWE method is between 155 g to 561 g and the R^2^ value is 0.95. Moreover, underwater fish weight estimation was exploited in Ref. [43]. A unidirectional tunnel controlled underwater studio was established by using a single camera. A fish is assumed to be positioned along the x-axis. A combination of 2D saliency detection and morphological operators are used for fish segmentation. The curve estimation for length measurement from segmented images is estimated by using a third-degree polynomial regression on the fish mid-point. Several regression algorithms were investigated to compute the weight of the fish. The performance of the method from Ref. [43] obtained an R^2^ value of 0.97. Based on the current state-of-the art fish weight-estimation methods, a special camera or controlled environment are commonly required for collecting fish images. A CNNs approach were used to identify fish in images. A regression learning approach is applied to estimate the weight of the fish and the significant fish features related to its weight. Those methods were used in different scenarios. For the proposed TWE method, a single camera is required without any other controlled environment. The general CNNs and regression learning models are formulated in a similar process as the other famous methods. However, the TWE-DRL algorithm requires only three features, i.e., the age, length, and width of the fish.

The limitations of the underwater fish weight-estimation methods are mostly based on the requirement to have special cameras and/or a controlled environment for collecting fish images. A fish weight-estimation-based deep learning approach consumes high computational complexity, while the regression learning approach is mostly applied for the case of off-water weight estimation. On the other hand, the limitation of our proposed method is that it requires a priori information of the fish’s age. In addition, the turbidity of the water has influences on fish detection to a certain degree. This is evident in the obtained results presented in the experiments across the different weeks due to the biofloc. For future work, a pseudo-stereo image will be introduced for extracting the depth of the fish directly from a single channel image recording and this will be used to produce the depth estimation [44,45].

The computational complexity of the proposed algorithm can be represented by a big-O notation. The proposed method has two major components: Firstly, the Tilapia detection based on the deep learning method and secondly, the Tilapia weight estimation based on the regression methods. For a deep learning algorithm, the computational complexity of the proposed method is dominated by the number of iterations and the number of network layers corresponding to the number of input data. The computational complexity of a neural network [46,47] in FC is $O\left(n^{4}\right)$,O(n), $O\left(n^{2}\right)$, O(k∗n∗log(n)∗m), *k* where *n* denotes the number of neighbors, *m* is the number of training data, and represents the number of features [48]. The complexity of the deep learning algorithm causes a large number of model parameters, which leads to a large memory. Mask R-CNN architecture is comprised of three major components, i.e., the Backbone, Head, and Mask Branch.

Each RoI needs to be calculated separately, which is time-consuming. In addition, the number of feature channels after RoI pooling is large, which makes the two FC layers consume a lot of memory and potentially affects the computational speed. The number of ResNet-50 parameters varies based on the number of layers, which are presented in Table 8.

Therefore, in our proposed method, the fish detection using Mask R-CNN consumes the most computational time. However, Mask RCNN yields higher accuracy. Though, given the current GPU configuration, this computational complexity is relatively modest.

## 4. Conclusions

Fish monitoring in underwater environments remains a challenging task due to many factors, such as the dynamics of fish moving, lighting conditions, the quality of water, and background noise. The focus of the paper lies in developing a low-cost practical single sensor imaging system with deep and regression learning algorithms for the non-intrusive estimation of fish weight. The proposed method consists of a Tilapia detection step and Tilapia weight-estimation step. The Tilapia datasets are curated and contain two types of datasets, one for the estimation of the fish’s depth from the camera and another for the estimation of the fish’s physical dimensions. A low-cost off-the-shelf camera is used for recording the fish. The Tilapia detection model has been trained by the image datasets using deep neural network, Mask R-CNN, with transfer learning. The Tilapia weight-estimating models are based on regression learning that require only three features of the fish, the fish’s length and width, depth, and age. Three regression learning methods have been investigated for Tilapia weight estimation. The experimental results show that the proposed algorithm has remarkable efficiency in estimating Tilapia weight with a MAE of 40.78 g, R2 of 0.74, and an average weight error of only 30.30 (±23.09) grams in a turbid water environment, which shows the practicality of the proposed framework. The principal strength of the proposed method is the continuous extraction of only three fish’s features that results in less time-consuming training processes, and its ability to estimate the weight of Tilapia in turbid water using low-cost video recording. The proposed algorithm has been demonstrated to be highly amenable to real-world fish farms by using only low-cost video cameras without including other special sensors.

## Figures and Tables

**Table 1:** Independent data and dependent output of TDepE, TP2CME, and TWE models.

Models	Independent Data	Dependent Output
TDepE	age of fish (weeks)	actual depth (cm)
	length of fish (pixel)	
	width of fish (pixel)	
TP2CME	age of fish (weeks)	
	length of fish (pixel)	length of fish (cm),
	width of fish (pixel)	width of fish (cm)
	depth (cm)	
TWE	age of fish (weeks)	weight of fish (g)
	length of fish (pixel)	
	width of fish (pixel)	
	depth (cm)	
	length of fish (cm)	
	width of fish (cm)	

**Table 2:** The number of training and testing data.

Models/Data	Training Phase		Testing Data
	Training Data	Validating Data	
Tilapia Detection	2101	900	1286
Depth Estimation	1555	389	833
Pixel-to-CM estimation	1555	389	833
Tilapia weight estimation	1555	389	833

**Table 3:** AP scores on Tilapia dataset of Faster R-CNN, Mask R-CNN, RetinaNet, and YOLO.

Deep Learning Networks	AP	AP_50_	AP_75_
Faster R-CNN	67.04	98.50	90.19
Mask R-CNN	75.68	99.11	92.12
RetinaNet	60.53	98.17	83.56
YOLO	62.61	90.37	78.56

AP is averaged over all categories where AP represents IoU = 0.50:0.05:0.95 (primary challenge metric), AP_50_ denotes IoU = 0.50 (PASCAL VOC metric), and AP_75_ is IoU = 0.75 (strict metric).

**Table 4:** Performance of Tilapia Depth Estimation with LR, RFR, and SVR methods.

Regression Models	Average Error	S.D. Error	MAE	*R* ^2^	MAE Ratio
LR	0.90	11.65	9.56	0.41	23.32
RFR	−0.23	7.65	5.60	0.75	7.47
SVR	−0.23	21.02	4.04	0.87	4.64

**Table 5:** Three experimental cases for determining the regression method to TP2CME and TWE.

Cases	TP2CME	TWE	Abbreviations
Case 1	SVR	LR, RFR, SVR	SSL, SSR, SSS
Case 2	RFR	LR, RFR, SVR	SRL, SRR, SRS
Case 3	LR	LR, RFR, SVR	SLL, SLR, SLS

**Table 6:** MAE and *R*^2^ scores of nine experimental cases for determining the regression method to TP2CME and TWE.

Measurement	SSL	SSR	SSS	SRL	SRR	SRS	SLL	SLR	SLS
MAE	81.39	109.64	99.20	47.18	51.56	97.21	42.54	52.67	99.26
*R* ^2^	0.11	−0.69	−0.25	0.71	0.61	−0.076	0.70	0.61	−0.21
MAE ratio	739.91	−158.90	−396.80	66.45	84.52	−1279.08	60.77	86.34	−472.67

**Table 7:** Comparison Tilapia Weight Estimation of Proposed TWE-DRL method with seven area-based weight estimations.

Methods	W1	W2	W3	W4	W5	W6	W7	Proposed Method
MAE	65.65	52.81	56.48	55.88	54.61	53.74	54.21	42.54
R^2^	0.52	0.65	0.42	0.44	0.43	0.48	0.45	0.70
MAE ratio	126.25	81.25	134.48	127.00	127.00	111.96	120.47	60.77

**Table 8:** Computation and parameters of ResNet-50.

Layer Name	Conv. 1	Conv. 2	Conv. 3	Conv. 4	Conv. 5	Total
Computation (MFLOPs)	118.816	672.358	953.344	1389.273	732.720	3867
Params (M)	0.0009664	0.218	1.226	7.118	14.987	23.550

## Data Availability

The data presented in this study are available on request from the corresponding author.

## References

[1] Sampantamit T., Ho L., Lachat C., Sutummawong N., Sorgeloos P., Goethals P. (2020). Aquaculture Production and Its Environmental Sustainability in Thailand: Challenges and Potential Solutions. Sustainability.

[2] Wu J., Zhou Y., Yu H., Zhang Y., Li J. A Novel Fish Counting Method with Adaptive Weighted Multi-Dilated Convolutional Neural Network. Proceedings of the International Conference on Ubiquitous Computing and Communications.

[3] Rossi L., Bibbiani C., Fronte B., Damiano E., Lieto A.D. Application of a smart dynamic scale for measuring live-fish biomass in aquaculture. Proceedings of the IEEE International Workshop on Metrology for Agriculture and Forestry.

[4] Tolentino L.K.S., De Pedro C.P., Icamina J.D., Navarro J.B.E., Salvacion L.J.D., Sobrevilla G.C.D., Madrigal G.A.M. (2020). Weight Prediction System for Nile Tilapia using Image Processing and Predictive Analysis. Int. J. Adv. Comput. Sci. Appl..

[5] Bravata N., Kelly D., Eickholt J., Bryan J., Miehls S., Zielinski D. (2020). Applications of deep convolutional neural networks to predict length, circumference, and weight from mostly dewatered images of fish. Ecol. Evol..

[6] Konovalov D.A., Saleh A., Efremova D.B., Domingos J.A., Jerry D.R. Automatic Weight Estimation of Harvested Fish from Images. Proceedings of the 2019 Digital Image Computing: Techniques and Applications (DICTA).

[7] Sant’Ana D.A., Pache M.C.B., Martins J., Soares W.P., de Melo S.L.N., Garcia V., Weber V.A.D.M., Heimbach N.D.S., Mateus R.G., Pistori H. (2021). Weighing live sheep using computer vision techniques and regression machine learning. Mach. Learn. Appl..

[8] Mathapo M.C., Tyasi T.L. (2021). Prediction of Body Weight of Yearling Boer Goats from Morphometric Traits using Classification and Regression Tree. Am. J. Anim. Vet. Sci..

[9] Ruchay A.N., Kolpakov V., Kalschikov V.V., Dzhulamanov K.M., Dorofeev K.A. (2021). Predicting the body weight of Hereford cows using machine learning. IOP Conf. Ser. Earth Environ. Sci..

[10] Hussain M.S., Mm A., Hm Y., Us B. (2019). Estimation of body weight and dressed weight in different sheep breeds of karnataka. Int. J. Vet. Sci. Anim. Husb..

[11] Weber V.A.D.M., Weber F.D.L., Gomes R.D.C., Oliveira A.D.S., Menezes G.V., De Abreu U.G.P., Belete N.A.D.S., Pistori H. (2020). Prediction of Girolando cattle weight by means of body measurements extracted from images. Rev. Bras. De Zootec..

[12] Alom M.Z., Taha T.M., Yakopcic C., Westberg S., Sidike P., Nasrin M.S., Hasan M., Van Essen B.C., Awwal A.A.S., Asari V.K. (2019). A State-of-the-Art Survey on Deep Learning Theory and Architectures. Electronics.

[13] Wageeh Y., Mohamed H.E.-D., Fadl A., Anas O., ElMasry N., Nabil A., Atia A. (2021). YOLO fish detection with Euclidean tracking in fish farms. J. Ambient. Intell. Hum. Comput..

[14] Cheng R., Zhang C., Xu Q., Liu G., Song Y., Yuan X., Sun J. (2020). Underwater Fish Body Length Estimation Based on Binocular Image Processing. Information.

[15] Saleh A., Laradji I.H., Konovalov D.A., Bradley M., Vazquez D., Sheaves M. (2020). A realistic fish-habitat dataset to evaluate algorithms for underwater visual analysis. Sci. Rep..

[16] Knausgård K.M., Wiklund A., Sørdalen T.K., Halvorsen K.T., Kleiven A.R., Jiao L., Goodwin M. (2021). Temperate fish detection and classification: A deep learning based approach. Appl. Intell..

[17] Dohmen R., Catal C., Liu Q. (2021). Image-based body mass prediction of heifers using deep neural networks. Biosyst. Eng..

[18] Qin H., Li X., Liang J., Peng Y., Zhang C. (2016). Deepfish: Accurate underwater live fish recognition with a deep architecture. Neurocomputing.

[19] Wan S., Yeh M.-L., Ma H.-L. (2021). An Innovative Intelligent System with Integrated CNN and SVM: Considering Various Crops through Hyperspectral Image Data. ISPRS Int. J. Geo-Inf..

[20] Cang Y., He H., Qiao Y. (2019). An Intelligent Pig Weights Estimate Method Based on Deep Learning in Sow Stall Environments. IEEE Access.

[21] Gjergji M., Weber V.D.M., Silva L.O.C., Gomes R.D.C., de Araujo T.L.A.C., Pistori H., Alvarez M. Deep Learning Techniques for Beef Cattle Body Weight Prediction. Proceedings of the 2020 International Joint Conference on Neural Networks (IJCNN).

[22] Zhang B., Guo N., Huang J., Gu B., Zhou J. (2020). Computer Vision Estimation of the Volume and Weight of Apples by Using 3D Reconstruction and Noncontact Measuring Methods. J. Sens..

[23] Qiao Y., Kong H., Clark C., Lomax S., Su D., Eiffert S., Sukkarieh S. (2021). Intelligent perception for cattle monitoring: A review for cattle identification, body condition score evaluation, and weight estimation. Comput. Electron. Agric..

[24] He K., Zhang X., Ren S., Sun J. Deep Residual Learning for Image Recognition. Proceedings of the IEEE Conference on Computer Vision and Pattern Recognition.

[25] Xavier A.I., Villavicencio C., Macrohon J.J., Jeng J.-H., Hsieh J.-G. (2022). Object Detection via Gradient-Based Mask R-CNN Using Machine Learning Algorithms. Machines.

[26] Shu J.-H., Nian F.-D., Yu M.-H., Li X. (2020). An Improved Mask R-CNN Model for Multiorgan Segmentation. Math. Probl. Eng..

[27] Mahmoud A.S., Mohamed S.S., El-Khoribi R.A., Abdelsalam H.M. (2020). Object Detection Using Adaptive Mask RCNN in Optical Remote Sensing Images. Int. J. Intell. Eng. Syst..

[28] Lin Y., Jeon Y. (2002). Random Forests and Adaptive Nearest Neighbors.

[29] Parathai P., Tengtrairat N., Woo W.L., Abdullah M.A.M., Rafiee G., Alshabrawy O. (2020). Efficient Noisy Sound-Event Mixture Classification Using Adaptive-Sparse Complex-Valued Matrix Factorization and OvsO SVM. Sensors.

[30] Hu B., Gao B., Woo W.L. (2021). A Lightweight Spatial and Temporal Multi-feature Fusion Linked Self-Attention Network for Defect Detection. IEEE Trans. Image Processing.

[31] Wang K., Cheng L., Yong B. (2020). Spectral-Similarity-Based Kernel of SVM for Hyperspectral Image Classification. Remote Sens..

[32] Vaillant J., Clouet A., Alleysson D. (2018). Color correction matrix for sparse RGB-W image sensor without IR cutoff filter. Unconv. Opt. Imaging.

[33] Gedraite E.S., Hadad M. Investigation on the effect of a Gaussian Blur in image filtering and segmentation. Proceedings of the ELMAR-2011.

[34] Malik S., Soundararajan R. (2021). A low light natural image statistical model for joint contrast enhancement and denoising. Signal Process. Image Commun..

[35] Srinivas K., Bhandari A.K. (2020). Low light image enhancement with adaptive sigmoid transfer function. IET Image Process..

[36] Ancuti C.O., Ancuti C., Vleeschouwer C.D., Bekaert P. (2018). Color Balance and Fusion for Underwater Image Enhancement. IEEE Trans. Image Process..

[37] Bernacki J. (2020). Automatic exposure algorithms for digital photography. Multimed Tools Appl..

[38] Parathai P., Tengtrairat N., Woo W.L., Gao B. (2019). Single-Channel Signal Separation Using Spectral Basis Correlation with Sparse Nonnegative Tensor Factorization. Circuits Syst. Signal Process..

[39] Tengtrairat N., Woo W.L., Parathai P., Aryupong C., Jitsangiam P., Rinchumphu D. (2021). Automated Landslide-Risk Prediction Using Web GIS and Machine Learning Models. Sensors.

[40] Koh B.H.D., Woo W.L. (2019). Multiview Temporal Ensemble for Classification of Non-Stationary Signals. IEEE Access.

[41] Tengtrairat N., Woo W.L. (2015). Single-Channel Separation using Underdetermined Blind Method and Least Absolute Deviation. Neurocomputing.

[42] Tengtrairat N., Woo W.L. (2014). Extension of DUET to Single-Channel Mixing Model and Separability Analysis. Signal Process..

[43] Sanchez-Torres G., Ceballos-Arroyo A., Robles-Serrano S. (2018). Automatic Measurement of Fish Weight and Size by Processing Underwater Hatchery Images. Eng. Lett..

[44] Tengtrairat N., Gao B., Woo W.L., Dlay S.S. (2013). Single-Channel Blind Separation using Pseudo-Stereo Mixture and Complex 2-D Histogram. IEEE Trans. Neural Netw. Learn. Syst..

[45] Tengtrairat N., Woo W.L., Dlay S.S., Gao B. (2016). Online Noisy Single-Channel Blind Separation by Spectrum Amplitude Estimator and Masking. IEEE Trans. Signal Process..

[46] Laudani A., Lozito G.M., Fulginei F.R., Salvini A. (2015). On Training Efficiency and Computational Costs of a Feed Forward Neural Network: A Review. Comput. Intell. Neurosci..

[47] Thompson N.C., Greenewald K.H., Lee K., Manso G.F. (2020). The Computational Limits of Deep Learning. arXiv.

[48] Kearns M.J. (1990). Computational Complexity of Machine Learning.

